# miR-98-5p contributes to cisplatin resistance in epithelial ovarian cancer by suppressing miR-152 biogenesis via targeting Dicer1

**DOI:** 10.1038/s41419-018-0390-7

**Published:** 2018-04-18

**Authors:** Yanan Wang, Wei Bao, Yuan Liu, Shiyu Wang, Shengjie Xu, Xi Li, Yanli Li, Sufang Wu

**Affiliations:** Department of Obstetrics and Gynecology, Shanghai General Hospital, Shanghai Jiao Tong University School of Medicine, Shanghai, China

## Abstract

Epithelial ovarian cancer (EOC) is a highly lethal gynecological malignancy, and cisplatin resistance is usually correlated with the poor prognosis of EOC. Increasing evidence indicates that the dysregulation of miRNAs is related to chemotherapy sensitivity. In this study, we revealed that miR-98-5p, a member of the let-7 family, was enriched in cisplatin-resistant EOC cells compared with cisplatin-sensitive cells, and could promote cisplatin resistance in EOC cells. Further studies showed that miR-98-5p could directly target the 3′-UTR of Dicer1 and suppress its expression, causing global miRNA downregulation. By miRNA array and qRT-PCR verification, we identified miR-152 as the vital downstream target of the miR-98-5p/Dicer1 axis in EOC cells. Moreover, we demonstrated that the ectopic expression of miR-152 reversed cisplatin resistance both in vitro and in vivo by targeting RAD51, a central member in homologous recombination. Importantly, miR-98-5p expression, as determined by in situ hybridization in tumor tissues, was associated with poor outcome of EOC patients. Together, these findings suggest the essential role of the miR-98-5p/Dicer1/miR-152 pathway in regulating cisplatin resistance of EOC cells and provide a potential target for EOC therapy.

## Introduction

Epithelial ovarian cancer(EOC) is the leading cause of deaths from gynecological malignancy in the developed world^1^. Due to the absence of specific symptoms in the early stages and the heterogeneous nature of this disease, more than two-thirds of patients cannot be diagnosed until an advanced stage^2^. The current standard treatment in patients with EOC is debulking surgery followed by platinum-based chemotherapy. However, ~25% of patients will develop resistance within 6 months after platinum-based chemotherapy^3^. The overall five-year survival rate for patients with advanced EOC is only 30–40%, and acquired resistance to platinum is considered a major factor in disease relapse. Therefore, it is crucial to investigate the mechanisms of platinum resistance in EOC patients and develop new strategies for EOC treatment.

MicroRNAs (miRNAs) are a class of non-protein-coding RNAs(~22 nt) that can act as post-transcriptional regulators by binding to the 3′-untranslated region(3′-UTR) of target mRNAs. Functional mature miRNAs arise from several post-transcriptional processing steps including cutting by Drosha/DGCR8 to pre-miRNA in the nucleus, exporting to the cytoplasm, and cleaving by the RNaseIII Dicer^4–6^. Several studies have shown a global decrease of mature miRNA expression in cancer cells, suggesting that miRNA biogenesis might be impaired in human cancers^7,8^. Additionally, low Dicer expression level has been significantly associated with advanced tumor stage and poor clinical outcomes among patients with EOC^9^. However, the underlying mechanisms or the biological advantages afforded to cells by reduced miRNA expression in cancers remains not fully illustrated.

The let-7 family is one of the earliest and most classic mammalian miRNAs identified^10,11^. The let-7 family is comprised of 13 family members located on 9 different chromosomes whose expression in most human malignancies is usually deregulated, reduced, or lost^12^. Importantly, it has been reported that Dicer1 is a direct target of let-7, which impacts the expression of other miRNAs^13^.

Previous findings identified miR-98-5p, a member of let-7 family, as a potent tumor suppressor downregulated in various cancer types, such as nasopharyngeal carcinoma^14^ and endometrial cancer^15^. However, miR-98 was also found to be upregulated in primary breast cancer specimens^16^, and expressed at higher levels in small cell lung cancer cell lines than immortalized human bronchial epithelial cells^17^. These results suggest that miR-98 may display absolutely contrary function in different types of cancers. However, few studies report the function of miR-98-5p in EOC.

In the present study, we identified miR-98-5p, a member of let-7 family, whose expression is associated with cisplatin resistance and poor outcome in EOC patients. Elevated miR-98-5p significantly promoted resistance of EOC cells to cisplatin treatment. Moreover, enforced miR-98-5p expression inhibited the expression of Dicer1, causing global miRNA downregulation. Specifically, by miRNA array, we identified miR-152 as the vital downstream target of the miR-98-5p/Dicer1 axis in EOC cells. Although a considerable amount of evidence indicates that miR-98 functions as a tumor suppressor, our data, for the first time, revealed that miR-98-5p could induce cisplatin resistance in EOC by suppressing the expression of miR-152 through directly targeting Dicer1. Our study provides a better understanding of the cisplatin resistance related mechanism in EOC, and offers miR-98-5p and miR-152 as potential therapeutic targets for cisplatin-resistant EOC.

## Results

### miR-98-5p expression in EOC

**Fig. 1:**
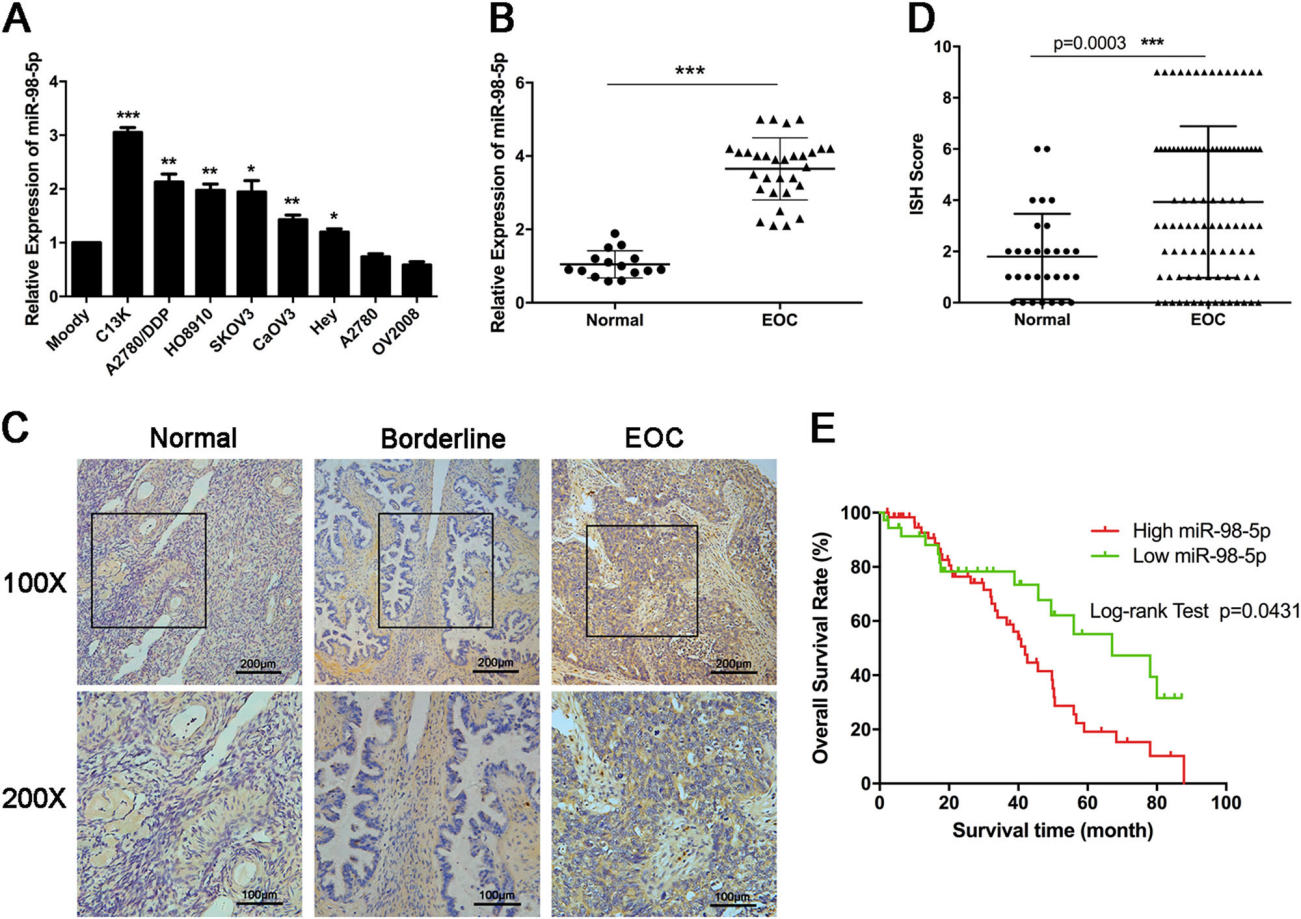
**Expression levels of miR-98-5p in EOC**. **a** Quantitative real-time PCR (qRT-PCR) analysis of miR-98-5p levels in Moody and 8 EOC cell lines. **b** Relative expression of miR-98-5p was assessed in fresh normal ovarian epithelial tissues and EOC tissues. The value was normalized to U6. **c** miR-98-5p expression was analyzed by in situ hybridization (ISH) in normal ovarian epithelial tissues (*n* = 30), borderline ovarian tumor tissues (*n* = 10), and EOC tissues (*n* = 97), and the representative images are shown. Scale bars, 200 μm (top) and 100 μm (bottom). **d** Scattergram analysis of the expression of miR-98-5p in normal ovarian epithelial tissues and EOC tissues. **e** A Kaplan–Meier analysis for overall survival of the 97 EOC patients with the corresponding expression of miR-98-5p is shown. *p < 0.05; **p < 0.01; ***p < 0.001

To investigate the role of miR-98-5p in the pathogenesis of EOC, we first examined its expression in EOC cell lines and clinical tissue specimens. We performed qRT-PCR to test the expression level of miR-98-5p in an immortalized human ovarian epithelial cell line (Moody) and eight EOC cell lines. The results demonstrated that the six EOC cell lines produced a greater amount of miR-98-5p than Moody (Fig. 1a). To verify the roles of miR-98-5p in clinical samples, we also detected endogenous expression levels of miR-98-5p in fresh normal ovarian epithelial tissues (*n* = 15) and EOC tissues (*n* = 30) using qRT-PCR (Fig. 1b). The expression levels of miR-98-5p were remarkably higher in EOC tissues than those in normal epithelial tissues. Furthermore, we determined the levels of miR-98-5p expression by in situ hybridization in another series of 97 EOC patients, for which clinical and prognosis data were available. The results showed that miR-98-5p was highly expressed in EOC tissues relative to normal tissues and borderline tumor tissues (Fig. 1c, d). We then analyzed the association between miR-98-5p and the clinic-pathological parameters of these EOC patients (Table 1). We found that expression of miR-98-5p was not statistically associated with age, stage, histology type, grade, or lymph node metastasis. However, high miR-98-5p expression was associated with poor therapy response and platinum resistance. Moreover, Kaplan–Meier analysis indicated a significant correlation between high miR-98-5p expression and poor overall survival (OS) of EOC patients (Fig. 1e).

**Table 1:** The clinic-pathological characteristics of EOC patients and association with miR-9 expression

Variables	Total	miR-98-5p		*P*-value
	(*n* = 97) No.	Low(*n* = 36) No. (%)	High (*n* = 61) No. (%)	
Age				
<50	42	17 (47.2)	25 (41.0)	0.5491
≥50	55	19 (52.8)	36 (59.0)	
FIGO stage				
I-II	30	13 (36.1)	17 (27.9)	0.3962
III-IV	67	23 (63.9)	44 (72.1)	
Histology type				
Serous	62	23 (63.9)	39 (63.9)	0.9865
Mucinous	22	8(22.2)	14 (23.0)	
Clear cell	6	2 (5.6)	4 (6.6)	
Endometrioid	7	3 (8.3)	4 (6.6)	
Differentiation				
G1	46	20 (55.6)	26 (42.6)	0.4508
G2	27	8 (22.2)	19 (31.2)	
G3	24	8 (22.2)	16 (26.2)	
Lymph nodes				
Negative	45	13 (36.1)	32 (52.5)	0.1188
Positive	52	23 (63.9)	29 (47.5)	
Response to primary therapy				
CR	66	29 (80.6)	37 (60.7)	0.0423*
Non-CR	31	7 (19.4)	24 (39.3)	
Platinum status				
Sensitive	58	27 (75.0)	31 (50.8)	0.0190*
Resistant	39	9 (25.0)	30 (49.2)	

Platinum resistance or sensitivity was defined as relapse or progression within 6 months or after 6 months from the last platinum-based chemotherapy, respectively.

*CR* complete response

* *p* < 0.05

Taken together, these data confirmed the overexpression of miR-98-5p in EOC, which is associated with platinum resistance and predicts poor prognosis in EOC patients.

### Roles of miR-98-5p and other members in let-7 family in cisplatin sensitivity of EOC cells

**Fig. 2:**
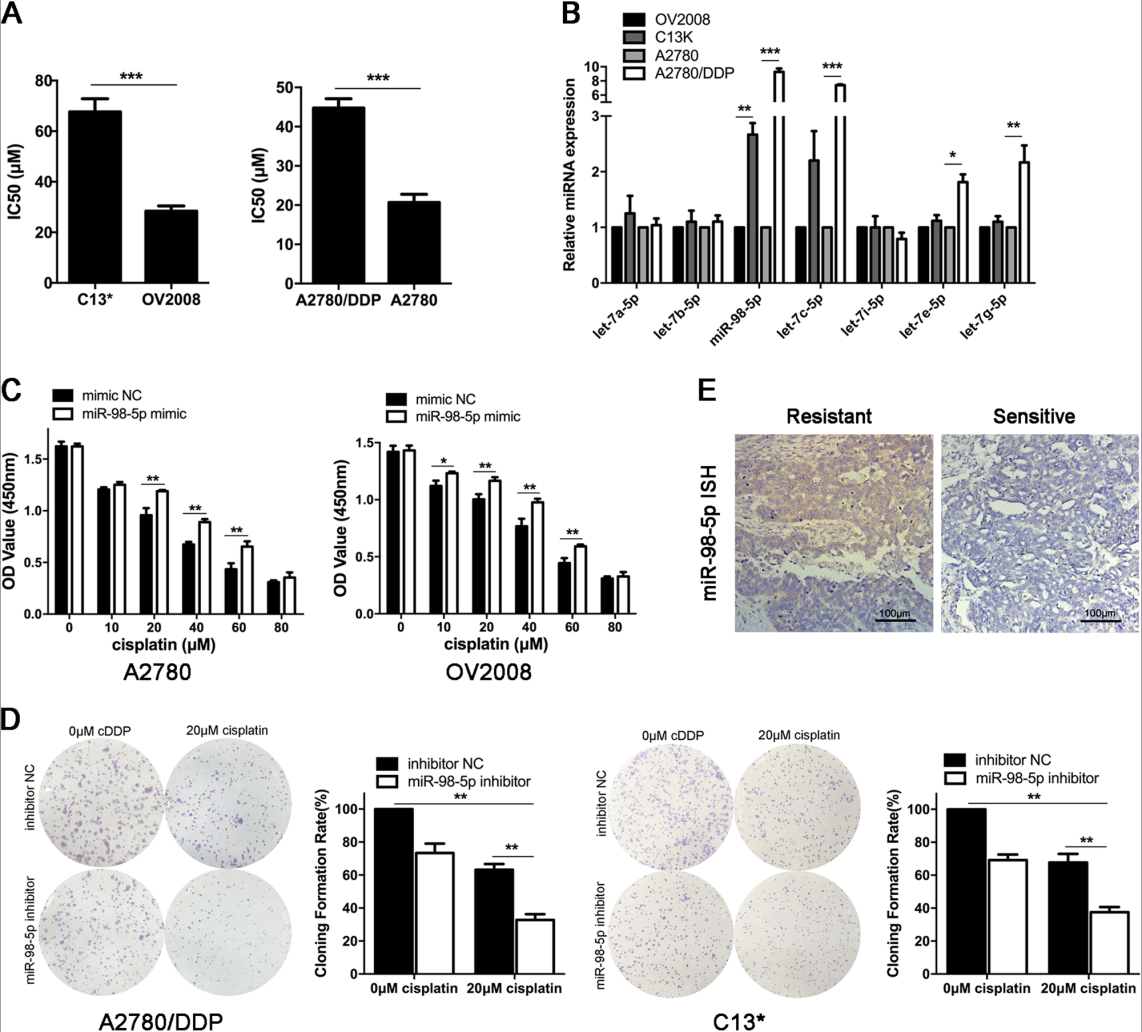
**Roles of miR-98-5p and other let-7 family members in cisplatin sensitivity of EOC cells.** **a** C13*, OV2008, A2780/DDP, and A2780 cells were exposed to various concentrations of cisplatin for 48 h, and the IC50 of cisplatin was examined by the CCK-8 assay. **b** Relative expression of let-7 family members in indicated cells were determined by qRT-PCR. **c** A2780 and OV2008 cells were transfected with mimic NC or miR-98-5p mimic for 24 h, and then cell viability was assayed after treatment with increasing concentrations of cisplatin for 48 h by CCK-8 assay. **d** Colony formation of C13* and A2780/DDP cells transfected with inhibitor NC or miR-98-5p inhibitor after exposure to cisplatin (20 μM). **E** Ovarian cancer tissues were incubated with anti-miR-98-5p probe for in situ hybridization (ISH). Representative images are shown. Scale bars, 100 μm. Each experiment was repeated three times. *p < 0.05; **p < 0.01; ***p < 0.001

We confirmed the cisplatin resistance of the EOC cell line C13* compared with the cisplatin-sensitive OV2008 cell line using CCK-8 assay. We found that the 50% inhibitory concentration (IC50) of cisplatin in the C13* cells was ~2.5-fold that of OV2008 cells (Fig. 2a). The experiment was repeated in another cisplatin-resistant EOC cell line A2780/DDP and its cisplatin-sensitive parental A2780. The results showed that the IC50 of A2780/DDP cells was two-fold that of A2780 cells (Fig. 2a). Then, we performed qRT-PCR to measure the expression levels of 7 members of the let-7 family in chemo-resistant and chemo-sensitive EOC cells. Let-7c-5p, let-7e-5p, and let-7g-5p were increased only in A2780/DDP cells, whereas miR-98-5p was upregulated in both C13* and A2780/DDP cells. The expression of the remaining miRNAs was similar in chemo-resistant and the relative chemo-sensitive cells (Fig. 2b).

Furthermore, to investigate roles of miR-98-5p in cisplatin-induced cytotoxicity, A2780 and OV2008 cells were transiently transfected with miR-98-5p mimic, and then exposed to different concentrations of cisplatin for 48 h and assessed by CCK8 assay. We found that miR-98-5p overexpression markedly increased the cisplatin resistance of EOC cells (Fig. 2c). Meanwhile, miR-98-5p inhibitor transfected cells significantly repressed colony formation and cell viability with cisplatin treatment (Fig. 2d and Supplementary Figure 1A). It is worth noting that HO8910, SKOV3, CaOV3, and Hey cells are not cisplatin-resistant cell lines, and the increased sensitivity to cisplatin of the 4 cell lines after downregulating miR-98-5p is a relative change. The efficacy of miR-98-5p overexpression and inhibition were verified by qRT-PCR (Supplementary Figure 1B). In addition, Fig. 2e shows the representative photographs of miR-98-5p expression in cisplatin-sensitive and cisplatin-resistant EOC tissues with in situ hybridization method. These data indicated that miR-98-5p was overexpressed in cisplatin-resistant EOC cells and tissues, and the downregulation of miR-98-5p could promote the cisplatin sensitivity of EOC cells.

### Dicer1 is a direct target of miR-98-5p in EOC cells

Evidence has suggested that Dicer1 is a direct target of let-7 family^13^, and the downregulation of Dicer1 contributes to cisplatin resistance in EOC cells^18^. However, the abundance of miRNAs or their targets in different cell types may show opposite outcomes, and miRNAs may perform different functions in different cell types by regulating their target genes^19^. We transfected A2780 cells with 50 nM mimics of let-7 family members and found that these miRNAs significantly inhibited Dicer1 protein expression, with miR-98-5p showing the greatest suppression effect (Supplementary Figure 2). Furthermore, we observed that the downregulation of Dicer1 markedly increased the cisplatin resistance of EOC cells with CCK-8 assay (Supplementary Figure 3).

**Fig. 3:**
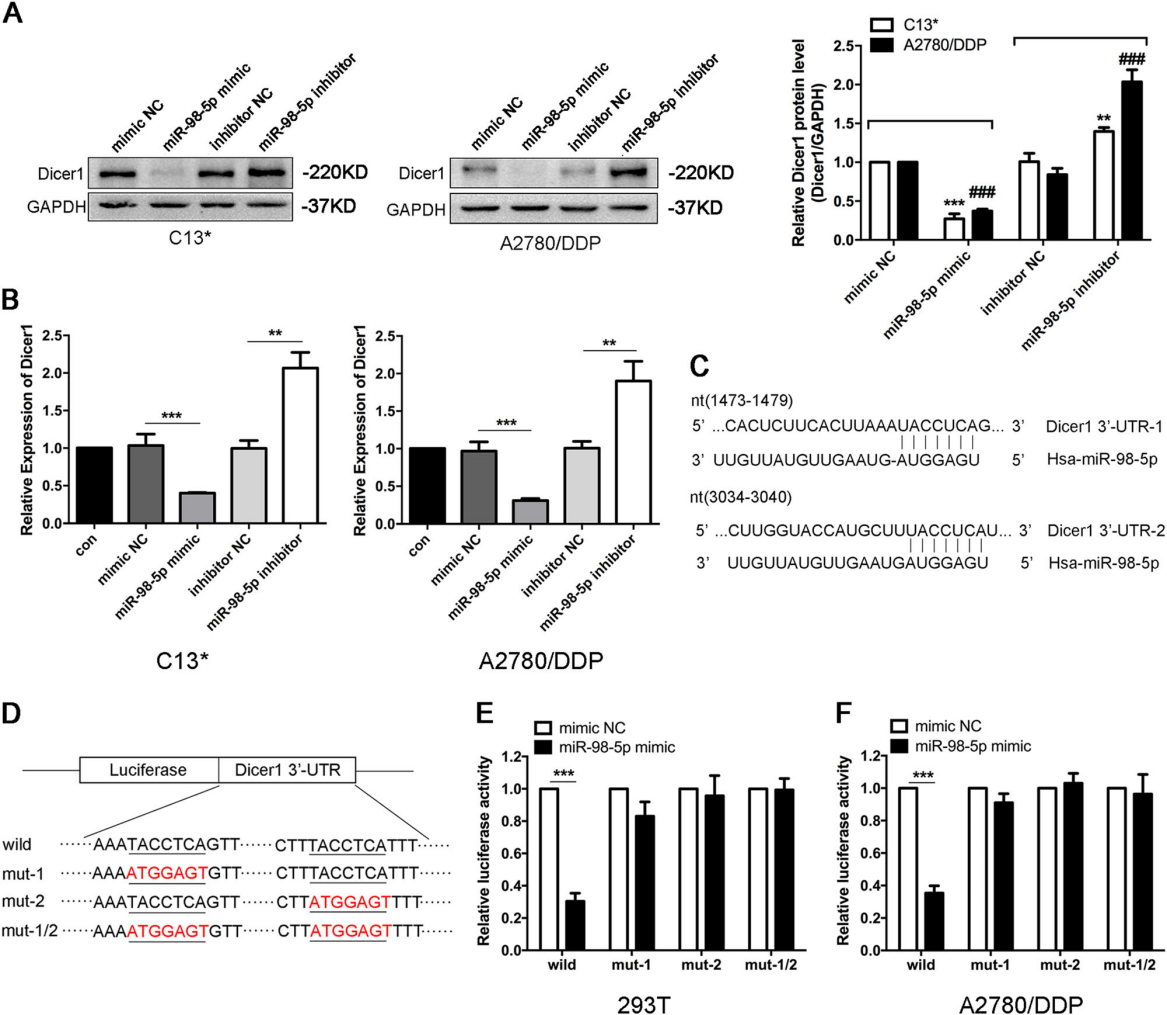
**Dicer1 is a direct target of miR-98-5p in EOC cells.** **a** Dicer1 protein levels in indicated cells were determined by western blot. **p < 0.01; ***p < 0.001 compared with C13* cells treated with mimic NC or inhibitor NC. ###*p* < 0.001 compared with A2780/DDP cells treated with mimic NC or inhibitor NC. **b** Dicer1 mRNA levels were determined by qRT-PCR after miR-98-5p overexpression or downregulation. **c** Diagram of predicted binding sites of miR-98-5p on the 3′-UTR of Dicer1gene. **d** Diagram of Dicer1 3′-UTR wild-type and mutant reporter constructs. **e**–**f** Luciferase reporter assays were performed in 293T and A2780/DDP cells with co-transfection of indicated wild-type or mutant 3′-UTR constructs and mimic NC or miR-98-5p mimic, respectively. Each experiment was repeated three times. **p < 0.01; ***p < 0.001

Next, we examined whether miR-98-5p, a member of let-7 family, could directly modulate Dicer1 and phenotypes in EOC cells. Two bioinformatics algorithms, TargetScan (http://www.targetscan.org) and miRanda (http://www.microrna.org/microrns/home.do), predicted that Dicer1 is a potential target of miR-98-5p. Indeed, we further observed that Dicer1 was significantly reduced at both mRNA and protein levels after miR-98-5p overexpression and, conversely, increased by the transfection of miR-98-5p inhibitor in both C13* and A2780/DDP cells (Fig. 3a, b). We then performed the dual-reporter luciferase assay in 293T cells to examine whether miR-98-5p could directly bind to the 3′-UTR of Dicer1. Transfection with miR-98-5p mimic reduced the luciferase activity of the construct, compared with mimic NC-transfected cells. However, upon site-directed mutagenesis of the putative miR-98-5p binding sites in these constructs (Fig. 3c, d), miR-98-5p lost the ability to downregulate luciferase activity (Fig. 3e, f).

Together, the data identified Dicer1 as a genuine target of miR-98-5p, which appeared to be involved in EOC development.

### miR-152 is markedly decreased in miR-98-5p/Dicer1 axis-regulating samples

**Fig. 4:**
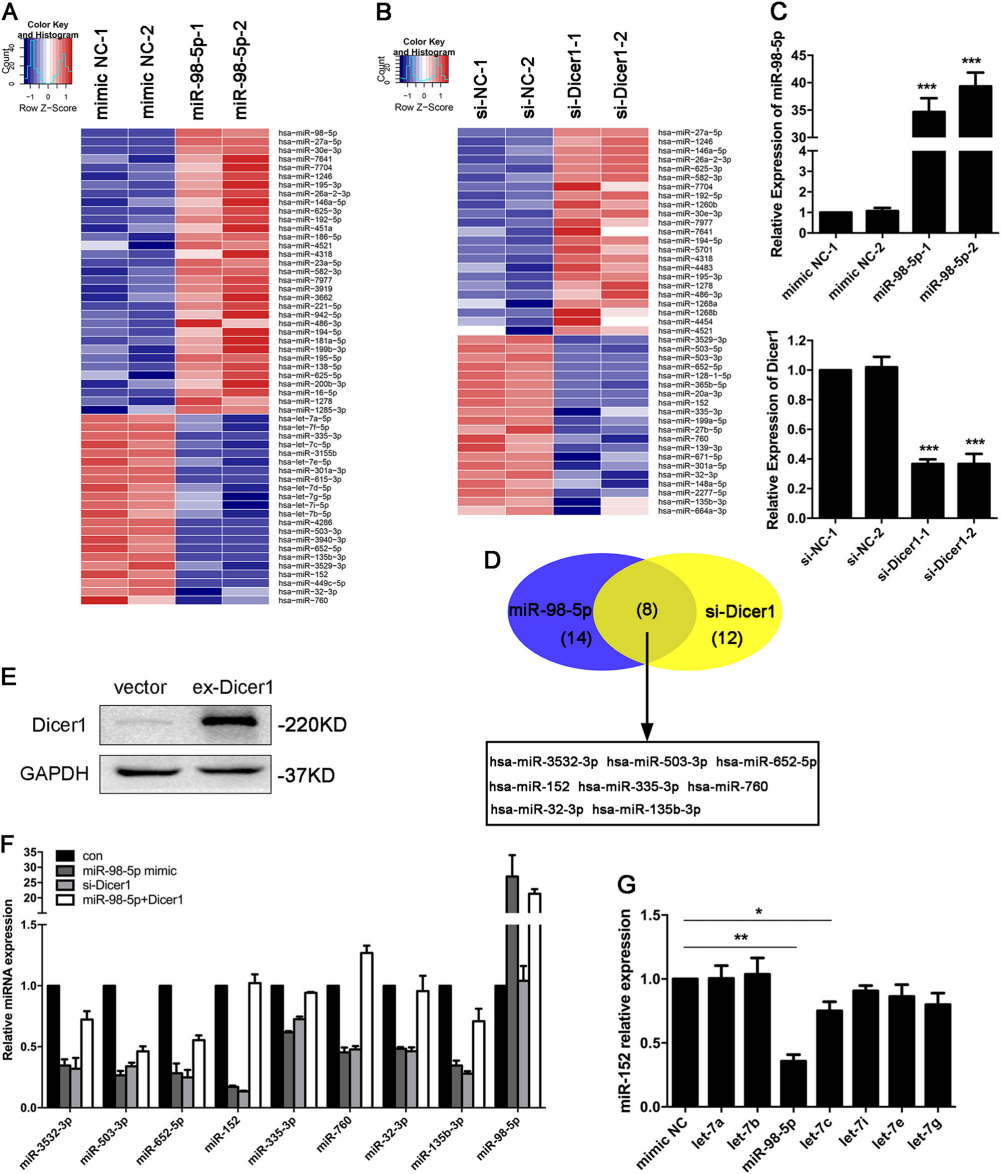
**miR-152 is markedly decreased in miR-98-5p/Dicer1 axis-regulating samples.** **a** A heatmap showing differentially regulated miRNAs in A2780 cells transfected with mimic NC or miR-98-5p mimic. A color scale is shown beside. **b** A heatmap showing differentially regulated miRNAs in A2780 cells transfected with si-NC or si-Dicer1. A color scale is shown beside. **c** The transfection efficiency was confirmed by qRT-PCR before miRNA sequencing. **d** Venn diagram showing the unique and overlapping miRNAs between miR-98-5p overexpressed and Dicer1 downregulated groups. **e**–**f** The selected miRNAs was confirmed by qRT-PCR analysis with total RNA isolated from indicated cells. **g** Quantitative RT-PCR analysis of miR-152 expression was performed in C13* cells transfected with let-7 family members. Each experiment was repeated three times. *p < 0.05; **p < 0.01; ***p < 0.001

We further sought to address the expression pattern of miRNAs in the downstream of miR-98-5p/Dicer1 axis. We conducted miRNA sequencing (Supplementary Excels) to identify differentially expressed miRNAs in miR-98-5p-transfected cells, and then a heatmap analysis was undertaken (Fig. 4a). The miRNA-sequence and analysis work was repeated in si-Dicer1-transfected cells (Fig. 4b). Additionally, the efficiency of transfection was determined by qRT-PCR analysis (Fig. 4c). Furthermore, we analyzed the downregulated miRNAs in miR-98-5p upregulated cells and dicer1 downregulated cells, and the Venn diagram revealed that 8 miRNAs were downregulated both in the two treatment groups (Fig. 4d).

To further confirm the robustness of the sequencing results shown above, we performed qRT-PCR assay to quantify the expressions of the 8 miRNAs in 4 treatment groups (Fig. 4e, f). Notably, miR-152 downexpression was most pronounced in miR-98-5p upregulated and Dicer1 downregulated cells compared with the negative control cells. Strikingly, the suppression effect of miR-98-5p on miR-152 was absolutely eliminated by the overexpression of Dicer1. In contrast, such an obvious change was not observed in the expression of the other 7 miRNAs. In addition, Fig. 4g shows that miR-152 is remarkably downregulated by miR-98-5p compared with the other members of the let-7 family. This result suggests that miR-152 is likely a vital and functional downstream regulator of the miR-98-5p/Dicer1 pathway.

### Effects of miR-152 on the cisplatin sensitivity of EOC cells in vitro and in vivo

**Fig. 5:**
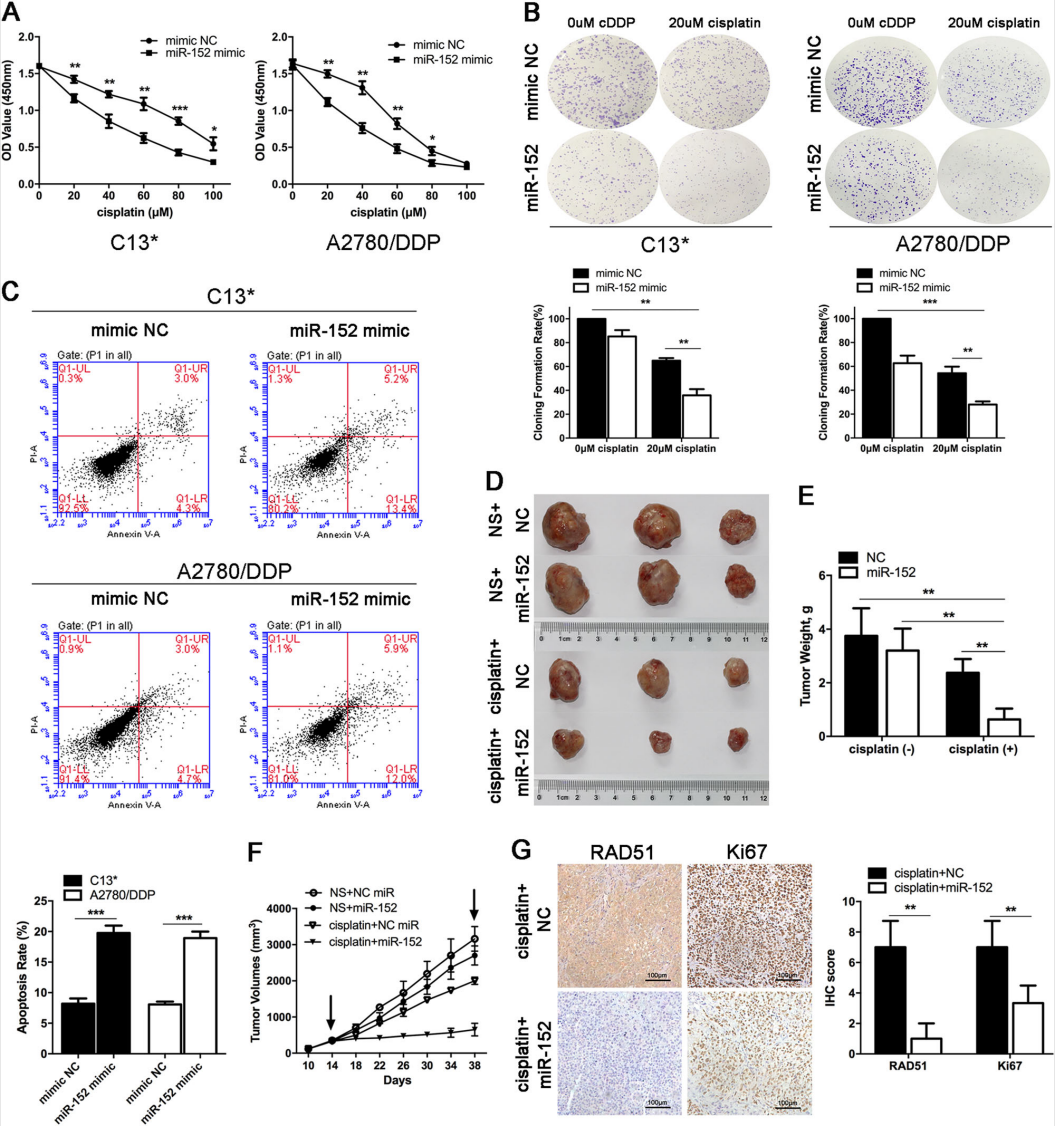
**Effects of miR-152 on cisplatin sensitivity of EOC cells in vitro and in vivo**. **a** C13* and A2780/DDP cells were transfected with mimic NC or miR-152 for 24 h, and then cell viability was assayed after treatment with increasing concentrations of cisplatin for 48 h by CCK-8 assay. **b** Colony formation of C13* and A2780/DDP cells transfected with mimic NC or miR-152 mimic after exposure to cisplatin (20 μM) **c** C13* and A2780/DDP cells transfected with mimic NC or miR-152 mimic were exposed to cisplatin (20 μM) for 48 h, and then the apoptotic cells were detected by flow cytometry. **d** The gross morphology of A2780/DDP cells subcutaneous xenograft tumors treated with NS plus agomiR-NC, NS plus agomiR-152, cisplatin plus agomiR-NC, or cisplatin plus agomiR-152 are shown. **e** Growth curves of xenograft tumors are shown. Tumor volumes were calculated as length × (square of width)/2. Arrows indicate the start and end of treatment, respectively. **f** The final xenograft tumor weights measured on day 38 after tumor cell injection. **g** The immunohistochemistry analyses for RAD51 and Ki67 were carried out on A2780/DDP xenograft tumor sections collected from mice with the indicated treatments. Representative staining are shown. Scale bars, 100 μm. *p < 0.05; **p < 0.01; ***p < 0.001

There has been a rapid increase in the number of studies focusing on miR-152 in recent years, which demonstrated that the expression of miR-152 was inhibited in various tumors, including ovarian^20^, hepatocellular^21^, and breast cancer^22^. These findings revealed that miR-152 may function as a tumor suppressor in human cancer. To further understand the role of miR-152 in cisplatin-induced cytotoxicity, miR-152 mimic was transfected into drug-resistant EOC cells C13* and A2780/DDP cells. The transfection efficiency was evaluated at 48 h after transfection (Supplementary Figure 4). The results by CCK-8 assay revealed that miR-152 overexpression sensitized C13* and A2780/DDP cells to cisplatin (Fig. 5a). Furthermore, we performed a colony formation assay and found that cells transfected with miR-152 mimic demonstrated reduced colony formation rates compared with NC after exposure to 20 μM cisplatin (Fig. 5b). We next performed a flow cytometry assay to investigate the function of miR-152 in cisplatin-induced apoptosis, and a higher proportion of apoptotic cells was observed in miR-152 overexpression cells compared with control cells (Fig. 5c). These data indicated that miR-152 could promote the cisplatin sensitivity of EOC cells.

To further demonstrate the role of miR-152 in chemo-sensitization, we used miR-152 agomiR, a cholesterol-conjugated 2′-O-methyl-modified miR-152 with pharmacokinetic properties, for in vivo studies^23^. According to the treatment, the tumors on the mice were actually assigned to the following groups: (1) agomiR-NC + NS, (2) agomiR-152 + NS, (3) agomiR-NC + cisplatin, and (4) agomiR-152 + cisplatin (Fig. 5d). As shown in Figs. 5e, f, the average xenograft volume and weight of agomiR-152 plus cisplatin group at 38 days were statistically significantly decreased compared with the other three groups. Moreover, immunohistochemistry assay showed that the tumors treated with agomiR-152 plus cisplatin displayed a decreased proliferation percentage of Ki-67 positive tumor cells compared with the control group (Fig. 5g). Taken together, these results demonstrated the reversion of cisplatin resistance by ectopic miR-152 expression in vivo.

### miR-152 expression is decreased in EOC, and miR-152 directly targets the 3′-UTR of RAD51 to decrease RAD51 expression

**Fig. 6:**
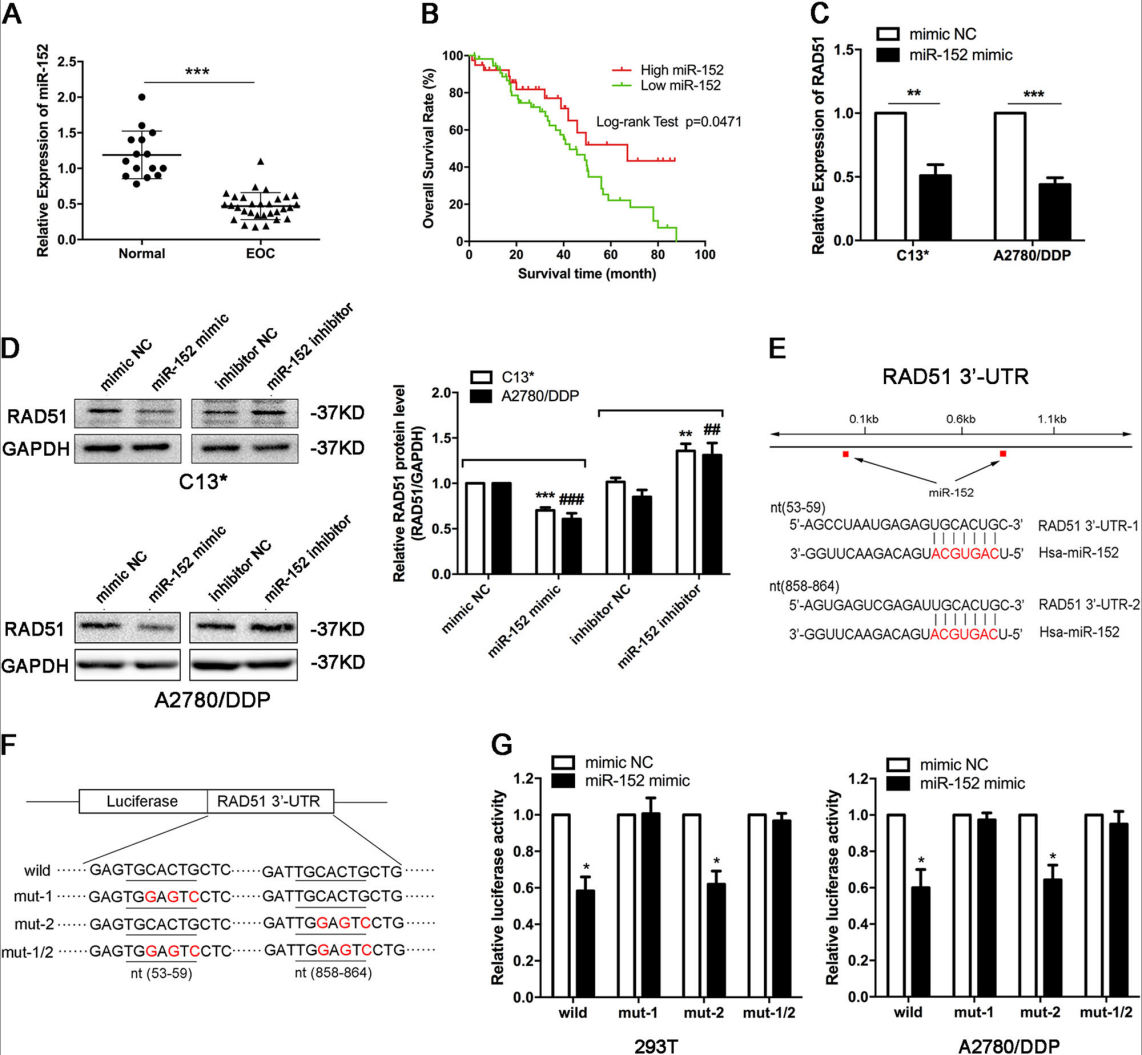
**miR-152 expression is decreased in EOC, and miR-152 directly targets the 3′-UTR of RAD51 to decrease RAD51 expression.** **a** Relative expression of miR-152 was assessed in fresh normal ovarian epithelial tissues and EOC tissues by qRT-PCR. The value was normalized to U6. **b** A Kaplan–Meier analysis for overall survival of the 97 EOC patients with the corresponding expression of miR-152 is shown. **c** mRNA expression of RAD51 in indicated cells were determined by qRT-PCR. **d** RAD51 protein levels in indicated cells were determined by western blot. **p < 0.01; ***p < 0.001 compared with C13* cells treated with mimic NC or inhibitor NC. ##*p* < 0.01; ###*p* < 0.001 compared with A2780/DDP cells treated with mimic NC or inhibitor NC. **e** Diagram of predicted binding sites of miR-152 on the 3′-UTR of RAD51 gene. **f** Diagram of RAD51 3′-UTR wild-type and mutant reporter constructs. **g** Luciferase reporter assays were performed in 293T and A2780/DDP cells with co-transfection of the wild-type or mutant 3′-UTR constructs and mimic NC or miR-152 mimic. Each experiment was repeated three times. *p < 0.05; **p < 0.01; ***p < 0.001

Subsequently, we detected the expression level of miR-152 in clinical tissues, and found that the expression of miR-152 was significantly weaker in EOC tissues than in normal tissues (Fig. 6a), and high miR-152 expression predicted better OS (Fig. 6b). This was consistant with the effect of miR-152 in turning around cisplatin resistance in EOC cells.

Next, we examined the mechanism by which miR-152 promote cisplatin sensitivity in EOC cells. With online prediction programs, we focused on RAD51, a central gene in homologous recombination (HR), which played a critical role in promoting strand transfer to resynthesize the damaged region^24^. Accordingly, we hypothesized that RAD51 may serve as a biologically relevant target of miR-152 and participate in miR-152-mediated regulation of chemo-resistance in EOC. In support of this concept, we transfected miR-152 mimic in EOC cells and found that the protein and mRNA levels of RAD51 were both significantly reduced compared with the control cells (Fig. 6c, d). Furthermore, two putative binding sites were identified within 3′-UTR of RAD51 and demonstrated as complementary to the seed sequences of the miR-152 gene (Fig. 6e). Indeed, the ectopic expression of miR-152 in 293T and A2780/DDP cells significantly repressed the luciferase activity of construct carrying the wild-type 3′-UTR sequence of RAD51 (Fig. 6f). Luciferase activity was also decreased when the binding site 2 was mutated, whereas mutation of the binding site 1 nearly rescued the decrease (Fig. 6g). These data suggest that miR-152 directly regulate RAD51 expression through its binding to site 1 (nt53–59) in the 3′-UTR of RAD51.

### miR-152 promotes the cisplatin sensitivity of EOC cells by targeting RAD51

**Fig. 7:**
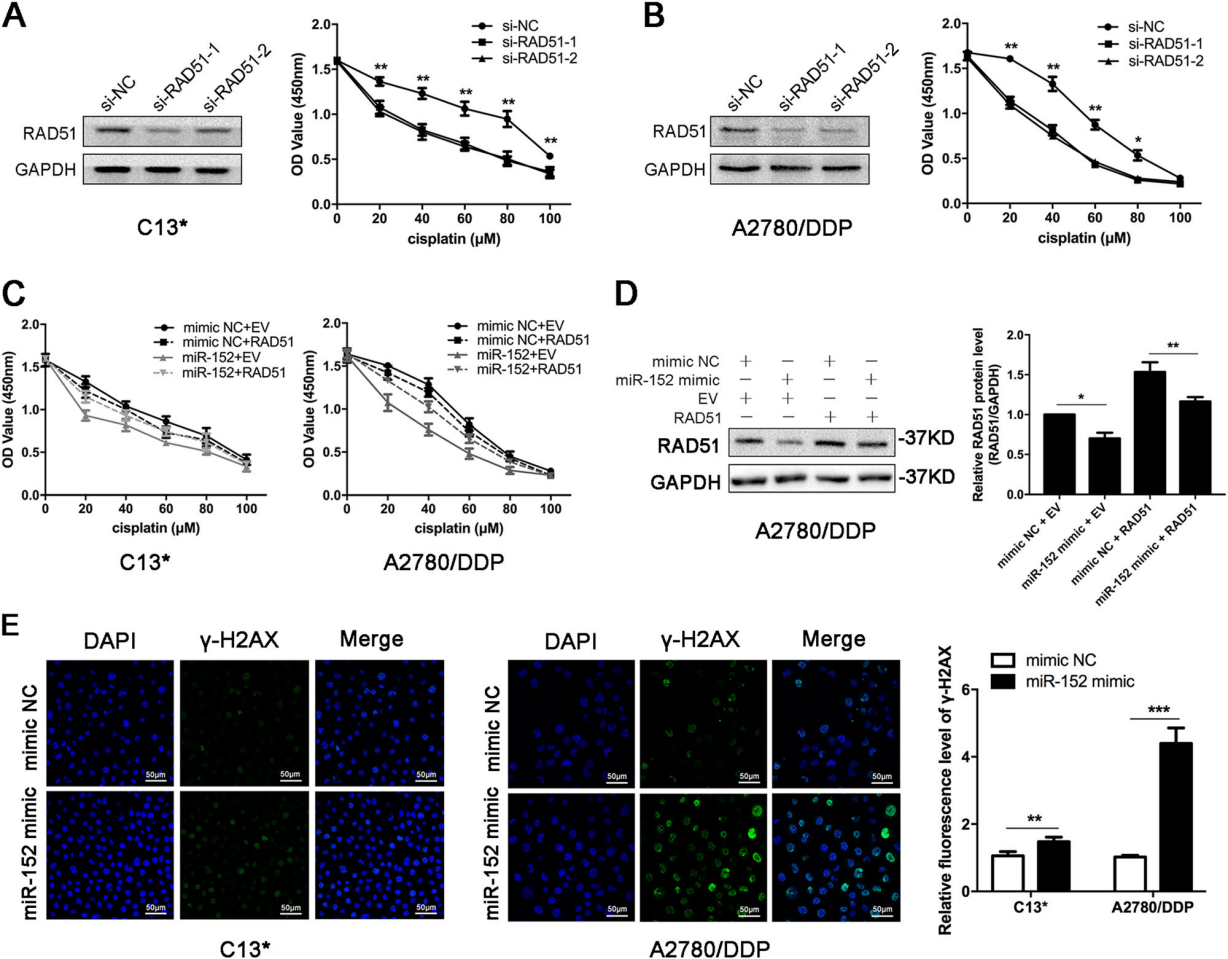
**miR-152 promotes cisplatin sensitivity of EOC cells by targeting RAD51.** **a**–**b** C13* and A2780/DDP cells were transfected with si-NC or si-RAD51 for 24 h, and then cell viability was assayed after treatment with various concentrations of cisplatin for 48 h by CCK-8 assay. **c** C13* and A2780/DDP cells were co-transfected with RAD51 without the 3′-UTR or empty vector (EV) together with mimic NC or miR-152 mimic for 24 h, and then cell viability was assayed after treatment with various concentrations of cisplatin for 48 h by CCK-8 assay. **d** Western blot analysis of A2780/DDP cells after transfection. **e** C13* and A2780/DDP cells were transfected with mimic NC or miR-152 mimic for 24 h. Then cells were treated with 20 μM cisplatin for 24 h, and γ-H2AX foci was examined by immunofluorescence. Scale bar, 50 μm. Each experiment was repeated three times. *p < 0.05; **p < 0.01

As RAD51 is an important protein in HR, knockdown of RAD51 could decrease DNA repair and increase sensitivity to DNA-damaging drugs, including cisplatin^25^. We further examined whether RAD51 suppression is critical for miR-152-induced cellular sensitivity to cisplatin in EOC cells. Remarkably, silencing RAD51 via small interfering RNAs (siRNA) statistically significantly sensitized C13* and A2780/DDP cells to cisplatin, similar to miR-152 mimic transfection (Fig. 7a, b). Moreover, the effect of miR-152 on cisplatin sensitivity was fully antagonized by the overexpression of RAD51 (by virtue of lacking the 3′-UTR), suggesting that miR-152-mediated sensitivity to cisplatin is primarily a result of RAD51 expression suppression (Fig. 7c, d).

Phosphorylated histone family member X (γ-H2AX), a key component in DNA repair, forms nuclear foci at sites of DNA damage and creates a focus for accumulation of members involved in DNA repair^26,27^. The γ-H2AX foci detected by IF were significantly higher in miR-152 transfected cells than the control cells after treating with cisplatin for 24 h (Fig. 7e).

These data confirmed that miR-152 regulated cisplatin sensitivity of EOC cells by targeting RAD51 and led to defects in HR.

### The miR-98-5p/Dicer1/miR-152/RAD51 pathway in EOC

**Fig. 8:**
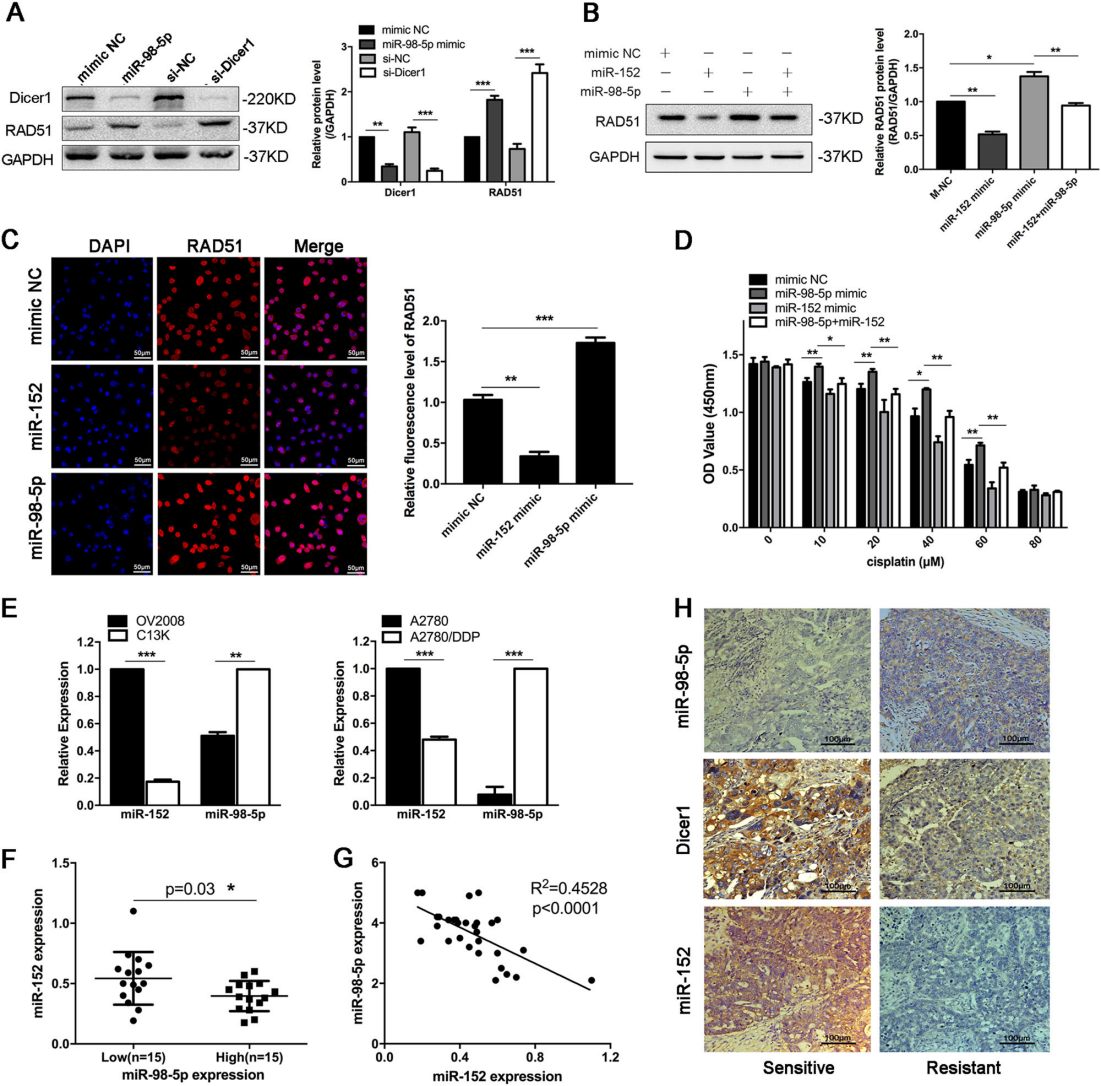
**The miR-98/Dicer1/miR-152/RAD51 pathway in EOC.** **a** The protein levels of Dicer1 and RAD51 in C13* cells were analyzed by western blot after transfection. **b** C13* cells were co-transfected with miR-152 mimic and miR-98-5p mimic. The protein levels of RAD51 were detected by western blot. **c** C13* cells were transfected with mimic NC, miR-152 or miR-98-5p mimic for 24 h, and then transfected cells were exposed to 20 μM cisplatin for 24 h, and RAD51 foci were examined by immunofluorescence. Scale bar, 50 μm. **d** After transfection, the change in cell response to an increasing concentration of cisplatin in C13* cells was examined by a CCK-8 assay. **e** qRT-PCR analysis of miR-152 and miR-98-5p expression in indicated cells. Each experiment was repeated three times. **f** EOC patients were stratified into miR-98-5p high and low expression according to mean of miR-98-5p expression, and miR-152 expression showed considerable differences between miR-98-5p low and high groups. **g** A plot of the relative expression of miR-98-5p vs miR-152 showed an inverse correlation between them. Correlation index was calculated using Spearman rank test (*R*^2^ = 0.4528, *p* < 0.0001). **h** Representative positive and negative staining of miR-98-5p, Dicer1, and miR-152 in human EOC tissue sections. Scale bar, 100 μm. *p < 0.05; **p < 0.01; ***p < 0.001

Rad51 is involved in HR repair, and relocalized in the nucleus in response to DNA damage. Therefore, the quantification of RAD51 foci could serve as a marker of HR. As shown in Fig. 8a, the upregulation of miR-98-5p or knockdown of Dicer1 significantly increased the expression of RAD51. However, miR-152 inhibited RAD51 expression in A2780/DDP cells, and upregulation of miR-152 abolished the ability of miR-98-5p to upregulate RAD51 level (Fig. 8b). The change of protein expression of RAD51 was further confirmed by immunofluorescence (Fig. 8c). In addition, to further study the roles of miR-152 in miR-98-5p-mediated cisplatin resistance, we co-transfected miR-98-5p mimic and miR-152 mimic in A2780 cells and found that miR-152 re-expression attenuated miR-98-5p-induced cell resistance to cisplatin by a CCK-8 assay (Fig. 8d). These results indicate that miR-152 functions as a mediator of miR-98-5p-induced RAD51 upregulation.

Next, we evaluated the expression relevance of miR-98-5p and miR-152 in EOC cells and tissues. The results revealed that miR-152 was significantly downregulated in cisplatin-resistant C13* and A2780/DDP cells, but miR-98-5p was overexpressed in C13* and A2780/DDP cells (Fig. 8e). Meanwhile, the protein level of RAD51 was lower in OV2008 and A2780 cells compared with C13* and A2780/DDP cells, which was consistant with their sensitivity to cisplatin (Supplementary Figure 5A). In addition, using Spearman’s correlation analysis, a negative correlation, with *R*^2^ = 0.4528, was observed between miR-98-5p and miR-152 (*p* < 0.0001), suggesting the existence of miR-98-5p-dependent regulation of miR-152 (Fig. 7f, g). Moreover, representative positive and negative staining of miR-98-5p, Dicer1, and miR-152 in human EOC tissue sections is shown in Fig. 8h. Furthermore, we performed a negative experiment to assess if miR-98-5p has effect on cisplatin response of COV362 cells with BRCA1 mutation. The results indicated COV362 cells treated with miR-98-5p mimic caused no obvious change in cisplatin response compared with the control cells (Supplementary Figure 5B), providing further evidence that high miR-98-5p expression causes cisplatin resistance via RAD51, a central member in HR.

**Fig. 9:**
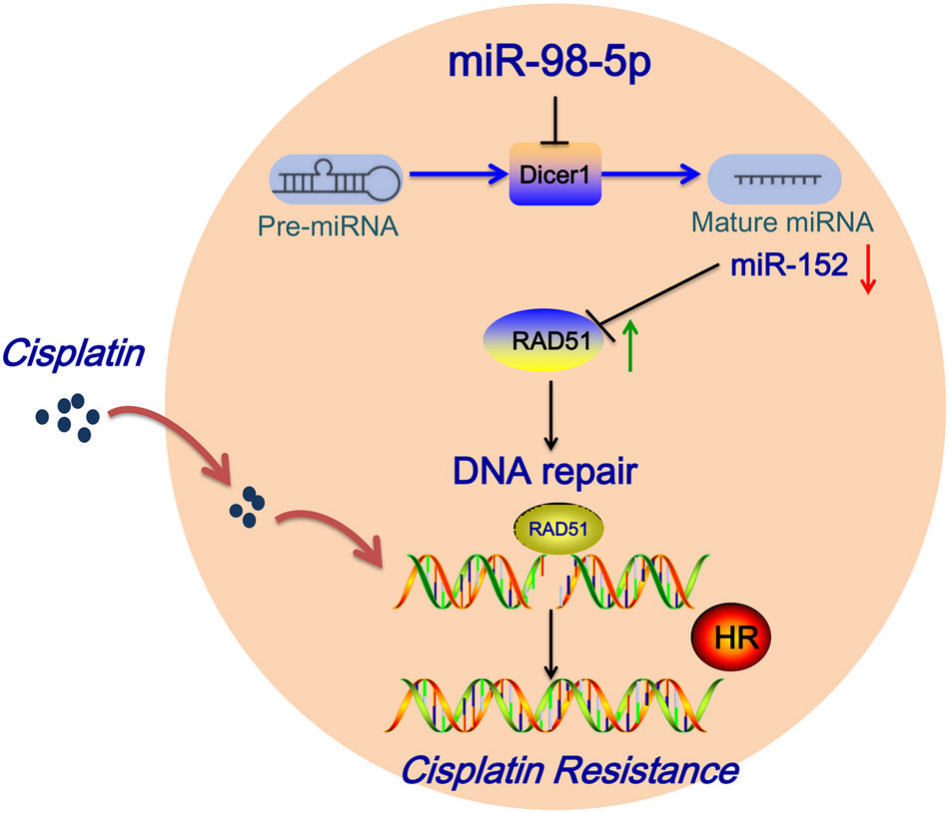
**The proposed working mode for the miR-98-5p-Dicer1-miR-152 pathway of chemo-resistance control in ovarian cancer.**

In conclusion, these results unveil a novel pathway driving chemo-resistance to cisplatin in EOC cells, where miR-98-5p inhibition of Dicer1 reduces the levels of miR-152, which binds to the 3′-UTR of RAD51 and enhances RAD51 expression, resulting in elevated HR efficiency (Fig. 9).

## Discussion

In clinical situations, acquired drug resistance frequently follows chemotherapeutic regimens and is considered the major cause of mortality in EOC^28^. Although cisplatin could cause intra-strand DNA crosslinks and is the first-line chemotherapy drug for ovarian cancer, drug resistance leading to chemotherapy failure is almost unavoidable. Increasing studies have suggested that miRNA is a new class of therapy molecule, and has a more modulatory role than traditional drugs.

A large body of evidence suggests that the global downregulation of miRNA expression is a widespread phenomenon in cancer^7,29,30^. The underlying mechanisms of miRNA dysregulation has not been fully illustrated or summarized. Epigenetic modifications, such as methylation, could directly modulate miRNA expression^31^. Additionally, transcription factors have been reported to play important roles in regulating miRNAs. For instance, transcription factor nrf2-regulated miR-1 and miR-206 to drive tumourigenesis^32^. Moreover, the regulation of miRNA processing factors also subsequently impacted miRNA expression and caused biological transformations in cancer. For example, Grazinao et al. identified that the miR-103/107 family inhibited the expression of Dicer, causing global miRNA downregulation and metastasis and poor outcome in breast cancer patients^33^.

The let-7 family has been known as one of the earliest miRNAs to be identified. It has been reported that let-7 could directly bind to the 3′-UTR of Dicer, and play a role in tuning of the mature miRNA expression and carcinogenesis. In the present study, the mechanism underlying the effects of miR-98-5p, a member of let-7 family, was explored in EOC. The upregulation of miR-98-5p was identified in EOC samples, and patients with high miR-98-5p expression had a significantly poorer prognosis than those with low miR-98-5p. We found that miR-98-5p could promote cisplatin resistance in EOC cells. Furthermore, our study demonstrated that miR-98-5p could bind to the 3′-UTR of Dicer1 and reduce the expression of Dicer1, which has been reported to associate with resistance to chemotherapy and poor clinical outcomes^9,34^. Then, we defined the identity of key miRNAs acting as the downstream mediators of the miR-98-5p/Dicer axis.

By miRNA sequencing, we screened out a subset of miRNAs downstream of the miR-98-5p/Dicer1 axis. We then focused on miR-152, because it was reduced most efficiently and previous studies have shown that this molecule displayed properties opposite of miR-98-5p in our study: miR-152 is downregulated in cisplatin-resistant EOC cells and contributes to cisplatin sensitivity in EOC^20^. Subsequently, the role of miR-152 in regulating EOC chemo-resistance was further confirmed by demonstrating RAD51 as a direct target of miR-152 and validating that miR-152 could induce defects in DNA repair and promote drug sensitivity to cisplatin in vitro and in vivo. HR is involved in tumor chemo-resistance, and RAD51 plays a primary role in this pathway. Indeed, dysregulation of RAD51 can sensitize tumor cells to DNA-damaging drugs, including cisplatin^25,35,36^. In addition, we disclosed miR-98-5p and miR-152 relevance in EOC, and concluded that miR-98/miR-152 relationship is a key player in the drug resistance of EOC cells.

However, it should be pointed out that the role of miR-98 in cancer is still controversial depending on the tumor type. Several studies have indicated that miR-98 serve as tumor suppressor, but the function of miR-98 is tumor-type specific. miR-98-5p was found to be downregulated in various cancer types^14,15^. Moreover, reduced miR-98-5p levels have also been implicated in resistance to cisplatin and radiotherapy, and the overexpression of miR-98-5p could promote chemosensitivity in lung adenocarcinoma^37^ and increase radio-sensitivity in esophageal squamous cell carcinoma^38^. However, miR-98 was also found to be upregulated in primary breast cancer specimens confirmed by real-time PCR and microarrays^16^, and expressed at higher levels in small cell lung cancer cell lines than in immortalized human bronchial epithelial cells^17^. These controversial observations indicate that the complexity of miRNAs and the function of specific miRNA differ markedly depend on tumor types.

The present study has several limitations. Although we observed that miR-98-5p could promote chemo-resistance to cisplatin in EOC cells, it might be better to use an in vivo model to further evaluate its effects. In addition, we could not address the question of why miR-98-5p and miR-103/107 regulate different downstream activities by targeting Dicer1 and display different functions in cancer. Other biological processes should be further explored.

This study focuses on miR-98-5p because it showed the greatest suppression effect on Dicer1 among the let-7 family and it was markedly upregulated in cisplatin-resistant EOC cells. Growing evidence suggests that the let-7 family plays a vital role in tumor suppression through repressing cell proliferation, invasion, metastasis, and resistance to therapy in lung cancer^39^, prostate cancer^40^, and hepatocellular carcinoma^41^. In the present study, we found that miR-152 is remarkably downregulated by miR-98-5p compared with other members of the let-7 family in EOC cells. This finding may explain the obviously different function of miR-98-5p from the let-7 family in EOC.

In conclusion, to our knowledge, we showed for the first time that miR-98-5p was upregulated in EOC samples, and miR-98-5p promoted chemo-resistance to cisplatin through a novel miR-98-5p/Dicer1/miR-152 pathway. In addition, our study showed that high miR-152 expression statistically significantly sensitized EOC cells to cisplatin in vitro and in vivo. These results may provide novel predictive and prognostic factors for EOC and the design of novel miRNA-based therapeutic strategies against EOC.

## Materials and Methods

### Cell culture and miRNA transfection

The human ovarian cancer cell lines C13*, OV2008, A2780, and its cisplatin-resistant cell line A2780/DDP were purchased from the American Type Culture Collection (Manassas, VA, USA). HO8910, SKOV3, CaOV3, Hey, COV362, and the immortalized ovarian epithelial cell line (Moody) were conserved in our laboratory. All cell lines were cultured in Dulbecco’s modified Eagle’s medium (Gibco, Auckland, New Zealand) supplemented with 10% fetal bovine serum and 1% penicillin/streptomycin in a humidified atmosphere of 5% CO_2_ at 37 °C. MiRNA mimic, inhibitor, and the negative controls were purchased from RiboBio (Guangzhou, China) and transfected into EOC cells with Lipofectamine 3000 (Life Technologies, Carlsbad, CA, USA) according to the manufacturer’s instructions.

### Gene silence and plasmid transfection

The Dicer1 and RAD51 siRNA were designed and synthesized by RiboBio (Guangzhou, China) to knock down the Dicer1 and RAD51, respectively. The target sequences are as follows: siDicer1: TGCTTGAAGCAGCTCTGGA and siRAD51: GACTGCCAGGATAAAGCTT. The Dicer1 plasmid and RAD51 plasmid (Table 2) were purchased from Qihe Biotechnology (Shanghai, China). The cells were transfected with siRNA or plasmid using Lipofectamine 3000 (Life Technologies), following the manufacturer's protocol.

**Table 2:** Cloning primers used in this study

Cloning primers	Sequence
DICER1 3′-UTR sense	5′-GGCGGCTCGAGGACTTGTAGGCACTCTTCAC-3′
DICER1 3′-UTR anti-sense	5′-AATGCGGCCGCGCAGGGTATCAGAATCTTT-3′
DICER1-mut-1 3′-UTR sense	5′-GGCGGCTCGAGGACTTGTAGGCACTCTTCAC-3′
DICER1-mut-1 3′-UTR anti-sense	5′-TTAAAATGGAGTGTTCTTCTTTTCTTTTGC-3′
DICER1-mut-2 3′-UTR sense	5′-CCATGCTTATGGAGTTTTCAAGAAAATATGCTT-3′
DICER1-mut-2 3′-UTR anti-sense	5′-TCTTGAAAACTCCATAAGCATGGTACCAAGTGC-3′
RAD51 3′-UTR sense	5′-AATTCTAGGCGATCGCTCGAGCGCCATTAATGCA GATGGAGT-3′
RAD51 3′-UTR anti-sense	5′-ATTTTATTGCGGCCAGCGGCCGCCTCACTCTGTCA CCCTGGCT-3′
RAD51-mut-1 3′-UTR sense	5′-TAAGTGCTGCAGCCTAATGAGAGTGGAGTCCTCC CTGGGGTTCTCTACAGGCCTC-3′
RAD51-mut-1 3′-UTR anti-sense	5′-GAGGCCTGTAGAGAACCCCAGGGAGGACTCCAC TCTCATTAGGCTGCAGCACTTA
RAD51-mut-2 3′-UTR sense	5′-GTGGAAGTTGCAGTGAGTCGAGATTGGAGTCCT GCATTCCAGCCAGGGTGACAGAGT-3′
RAD51-mut-2 3′-UTR anti-sense	5′-ACTCTGTCACCCTGGCTGGAATGCAGGACTCCA ATCTCGACTCACTGCAACTTCCAC-3′
PcDNA-Dicer1 sense	ATGAAAAGCCCTGCTTTGCAAC
PcDNA-Dicer1 anti-sense	CCTCAGGTTCCCAATAGCTGA
PcDNA-RAD51 sense	ATGGCAATGCAGATGCAGCTT
PcDNA-RAD51 anti-sense	AGTGGGAGATGCCAAAGACTGA

### EOC tissue samples

For formalin-fixed, paraffin-embedded samples, 30 normal ovarian tissues, 10 borderline tissues, and 97 EOC tissues were collected from patients at Shanghai Jiao Tong University Affiliated Shanghai General Hospital between 2004 and 2016. All patients were treated with standard care of platinum-based therapy after surgery, and the informed consent was obtained from all patients. Platinum sensitivity or platinum resistance was defined as relapse or progression within 6 months or after 6 months from the last chemotherapy, respectively. Primary therapy response was defined as the response evaluation criteria in solid tumors. We obtained ethical approval and support from the Institutional Research Ethics Committee of Shanghai Jiao Tong University Affiliated Shanghai General Hospital.

For fresh tissues, 15 normal ovarian tissues and 30 EOC tissues were gathered between June, 2014 and September, 2015, and stored at −80 °C. The tumor content of the tissues was examined by haematoxylin and eosin staining in the pathology units. Only specimens containing more than 60% of tumor tissue were used in this study.

### Quantitative real-time PCR

Total RNA from tumor samples or cell lines was extracted using TRIzol reagent (TaKaRa, Dalian, China). Complementary DNA was synthesized with random primers or miRNA specific stem-loop primers (RiboBio) using a PrimeScript RT reagent kit (TaKaRa). Subsequently, qRT-PCR was performed with SYBR Premix Ex Taq (TaKaRa) on a 7500 real-time PCR system (AB Applied Biosystems, Mannheim, Germany). The 2^−ΔΔCt^ method was applied to calculate the relative expression levels using GAPDH or U6 as the endogenous control. The mRNA primer sequences are listed in Table 3.

**Table 3:** qRT-PCR Primers used in this study

qRT-PCR Primers	Sequences
GAPDH sense	5′-GAAATCCCATCACCATCTTCCAGG-3′
GAPDH anti-sense	5′-GAGCCCCAGCCTTCTCCATG-3′
DICER1 sense	5′-GAGCTGTCCTATCAGATCAGGG-3′
DICER1 anti-sense	5′-ACTTGTTGAGCAACCTGGTTT-3′
RAD51 sense	5′-CAACCCATTTCACGGTTAGAGC-3′
RAD51 anti-sense	5′-TTCTTTGGCGCATAGGCAACA-3′

### Western blotting

Equal amounts of cellular protein extracts were separated on SDS-polyacrylamide mini-gels and transferred onto a PVDF membrane (Millipore, Billerica, MA, USA) at 350 mA for 1.5 h. The membranes were blocked with 5% BSA (Roche, Mannheim, Germany) for 1 h, and then incubated with the appropriate primary antibody overnight at 4 °C. Antibodies against human RAD51, Dicer1 were purchased from Abcam Inc. Antibodies against GAPDH was purchased from Cell Signaling Technology, Inc. After washing with Tris-buffered saline with Tween-20 (TBST) three times, the membranes were incubated with secondary antibody for 1 h at room temperature. The proteins were visualized using ECL chemiluminescence (Millipore), and quantified with ImageJ (National Institutes of Health, Bethesda, MD, USA).

### Colony formation assay

Twenty-four hours after transiently transfecting with miR-152, miR-NC, si-RAD51, or si-NC, the cells were harvested. The cells were seeded onto six-well plates at a density of 800 cells per well, treated with or without  20 μM cisplatin for 48 h, and then recovered for 10 days in fresh DMEM containing 10% FBS. The cells were fixed in methanol and stained with 0.1% crystal violet. The samples were photographed and the numbers of visible colonies that contained >50 cells were counted.

### In situ hybridization and scoring

The expression of miRNA in paraffin-embedded tissue specimens was determined by an in situ hybridization kit (MK1030, Boster, Wuhan, China). Briefly, 6-um-thick sections of paraffin-embedded specimens were deparaffinized with xylene and rehydrated in a series of ethanol. After Proteinase-K incubation for 15 min at 37 °C, the slides were prehybridized in a hybridization solution at 37 °C for 2 h. Then, tissue sections were hybridized with 5′-digoxigenin-labeled (DIG-labelled) oligonucleotide probe at 37 °C overnight. After stringent washes with 5 × SSC, 1 × SSC, and 0.2 × SSC buffers, the sections were blocked with DIG blocking buffer at 37 °C for 30 min. An anti-DIG antibody was applied, and the sections were incubated at 37 °C for 1 h. After washing in a staining solution, the sections were developed by diaminobenzidine-hydrogen peroxide. Scoring was measured by the cell cytoplasm staining. The sections were evaluated based on the percentage of positively stained cells (0–3) and the intensity of staining (0–3). Then, the score of miRNA expression was calculated as percentage × intensity of the staining. Therefore, score 0 presents negative (−), 1–2 as weak positive, (+), 3–4 as moderate positive (++), and 6–9 as strong positive (+++). Scoring with ‘−’ and ‘+’ was regarded as lower miR-98-5p expression, whereas ‘++’ and ‘+++’ represented higher expression of miR-98-5p.

### Cell immunofluorescence staining

Cells were grown on coverslips and exposed to  20 µM cisplatin for 24 h. After treatment, the cells were fixed with 4% paraformaldehyde for 20 min, blocked with 5% bovine serum albumin containing 0.2% Triton X-100 for 30 min, and incubated with antibodies against RAD51 (1:200, Abcam) or γ-H2AX (1:200, Cell Signaling Technology) overnight at 4 °C. Then, the cells were incubated with relative secondary antibodies conjugated to fluorescein isothiocyanate (1:100, Molecular Probes, Invitrogen, USA) for 1 h at room temperature. Nuclei were counterstained with 4′6-diamidino-2-phenylindole (DAPI) for 5 min. The fluorescence images were captured with laser-scanning confocal microscopy (Leica, Heidelberg, Germany).

### Apoptosis assay

For the assessment of apoptosis, the cells were collected and stained with the Annexin V Apoptosis Detection kit APC (eBioscience, San Diego, CA, USA). The flow cytometry events were conducted on the FACSCalibur flow cytometer. The data for apoptosis were analyzed with FlowJo (Tree Star, Ashland, OR, USA).

### Luciferase reporter assay

The 3′-UTR of RAD51 containing the wild-type or mutated binding sites of miR-152 were amplified with PCR method (Table 2). We generated four Luc-RAD51 3′-UTR constructs, including one wild-type binding sequence and three mutated binding sequences. All the constructs containing 3′-UTR inserts were sequenced and verified. The Luciferase assay was performed in 293T and A2780/DDP cell lines. Cells were seeded into 24-well plates in triplicate. After 24 h, the cells were transfected with RAD51 reporter constructs and miR-152 mimic, or miR-NC using Lipofectamine 3000 (Invitrogen). Luciferase activity was measured in cell lysates 24 h after transfection using a Dual Luciferase Reporter System (Promega, Madison, WI, USA).

### Chemosensitivity assay

Cisplatin was puechased from Sigma-Aldrich, US. Logarithmically growing cells were transfected with 50 nM miRNA mimics/inhibitors or miR NC, and then seeded onto 96-well plates at 1 × 10^4^ cells/well at 24 h later. After incubating in medium containing various concentration of cisplatin for 48 h, cell viability was estimated by the CCK-8 reagent (Dojindo, Kumamoto, Japan) at 450 nm using a Model 550 series microplate reader (Bio-Rad Laboratories). The assay was performed with three replicates. The IC_50_ values were then calculated.

### Tumor xenograft model

All of the animal experiments were performed in strict accordance with the Guide for the Care and Use of Laboratory Animals, and approved by the Department of Laboratory Animal Science at Shanghai Jiao Tong University School of Medicine. A total of 12 BALB/C female athymic mice at 4 weeks of age were housed and maintained in specific pathogen-free conditions. We subcutaneously injected 8 × 10^6^ A278/DDP cells resuspended in 100 µL of phosphate-buffered saline into the ambilateral flank of mice. 10 days later, the mice were randomly assigned into two groups with 6 mice per group. AgomiR-9 and agomiR-NC (RiboBio) were directly injected into the left and right flank tumor of the 12 mice, respectively, at a dose of 5 nmol (diluted in 50 µL phosphate-buffered saline) per mouse every 4 days for 8 times. After 4 days, the mice in the experiment group were treated with cisplatin at a dose of 5 mg/kg intraperitoneally every 4 days for 7 times, and the control group mice were injected with normal saline. After the initial treatment, the tumor size was assessed every 4 days by a digital caliper, and tumor volume was calculated as length × (square of width)/ 2. The mice were killed following cervical dislocation under anesthesia at 38 days after injection. The experiments were performed in an observer-blinded and randomized manner.

### Statistical analysis

All data are presented as the means ± SD. Two-tailed Student’s *t*-test was used to compare the two groups. Survival curves were examined by Kaplan–Meier analysis with the log-rank test analysis. Spearman’s non-parametric correlation test was performed to examine the correlation between expression levels of miR-98-5p and miR-152 by GraphPad Prism 5.0 (GraphPad Software, Inc., La Jolla, CA, USA). All statistical analyses were performed using SPSS 20.0. *P*-value < 0.05 was considered statistically significant.

## Electronic supplementary material

Supplementary figures and legends

miRNA sequencing data-mimic NC and miR-98-5p mimic

miRNA sequencing data-si-NC and siDicer1
