# Supplementary material for: miR-98-5p contributes to cisplatin resistance in epithelial ovarian cancer by suppressing miR-152 biogenesis via targeting Dicer1
Source: Cell Death Dis. 2018 Apr 18;9(5):447. doi: 10.1038/s41419-018-0390-7 (PMC5906447; doi:10.1038/s41419-018-0390-7)
Supplement: Supplementary file 1 — Supplementary figures and legends [file 41419_2018_390_MOESM1_ESM.docx]

Supplementary Figures


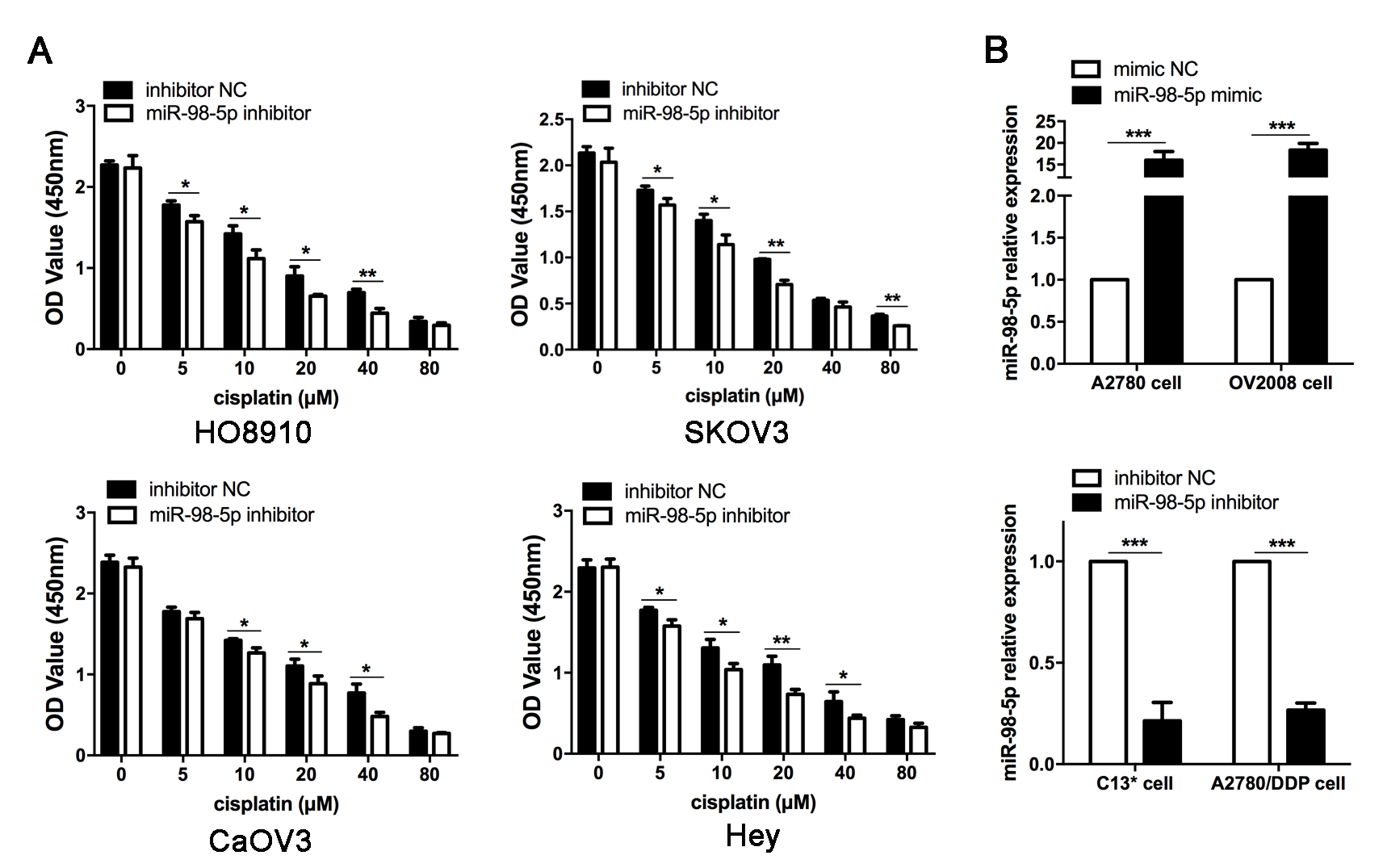


**Supplementary Figure 1**. (**A**) HO8910, SKOV3, CaOV3 and Hey cells were transfected with inhibitor NC or miR-98-5p inhibitor for 24 h, and then cell viability was assayed after treatment with increasing concentrations of cisplatin for 48 h by CCK-8 assay. (**B**) miR-98-5p relative expression in EOC cells transfected with NC, miR-98-5p mimic or miR-98-5p inhibitor for 48 h was determined by qRT-PCR. Each experiment was repeated three times. *p < 0.05; **p < 0.01; ***p < 0.001.


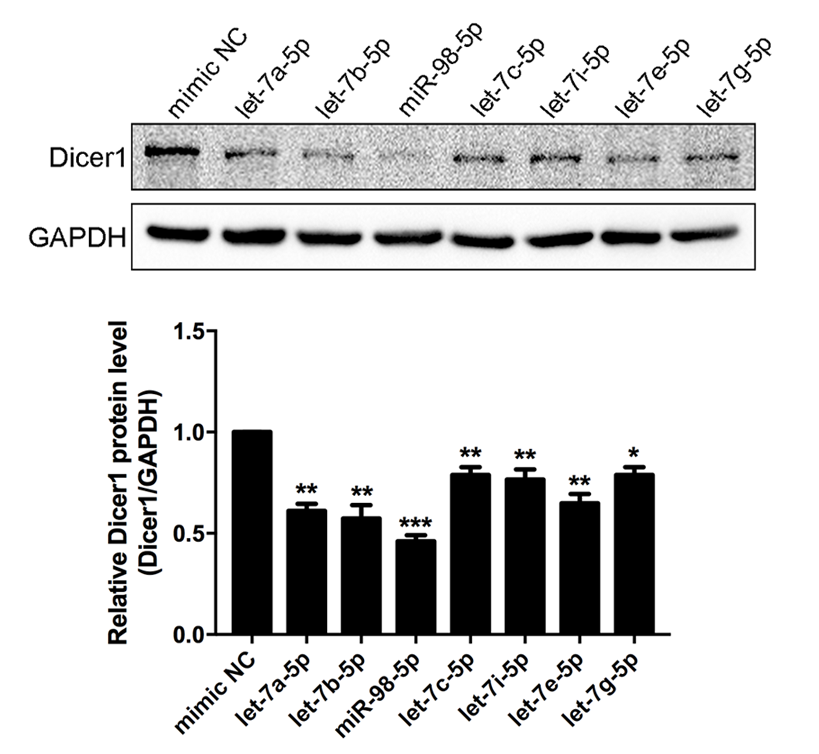


**Supplementary Figure 2**. The protein levels of Dicer1 in A2780 cells transfected with indicated miRNA were detected by western blot. The experiment was repeated three times. *p < 0.05; **p < 0.01; ***p < 0.001.


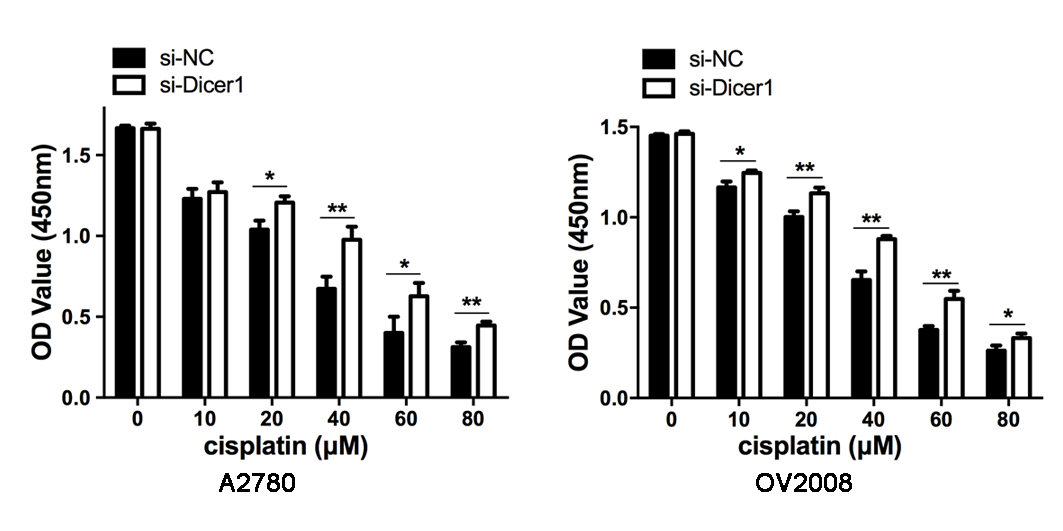


**Supplementary Figure 3**. A2780 and OV2008 cells were transfected with si-NC or si-Dicer1 for 24 h, and then cell viability was assayed after treatment with increasing concentrations of cisplatin for 48 h by CCK-8 assay. Each experiment was repeated three times. *p < 0.05; **p < 0.01.


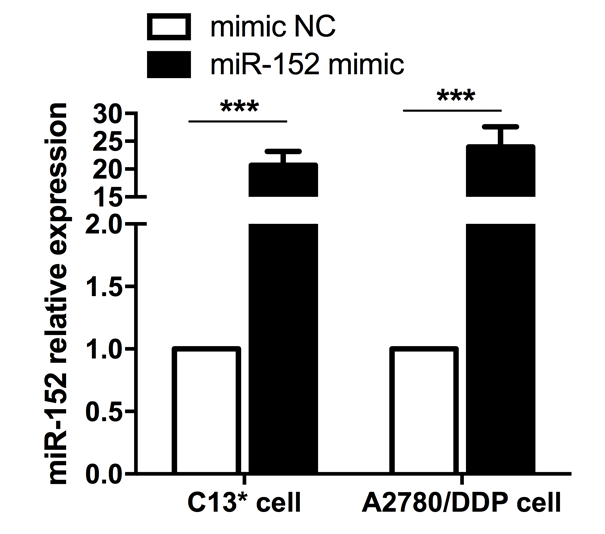


**Supplementary Figure 4**. The efficiency of miR-152 over-expression by transfection with miR-152 mimic was confirmed by qRT-PCR in C13* and A2780/DDP cells. The experiment was repeated three times. ***p < 0.001.


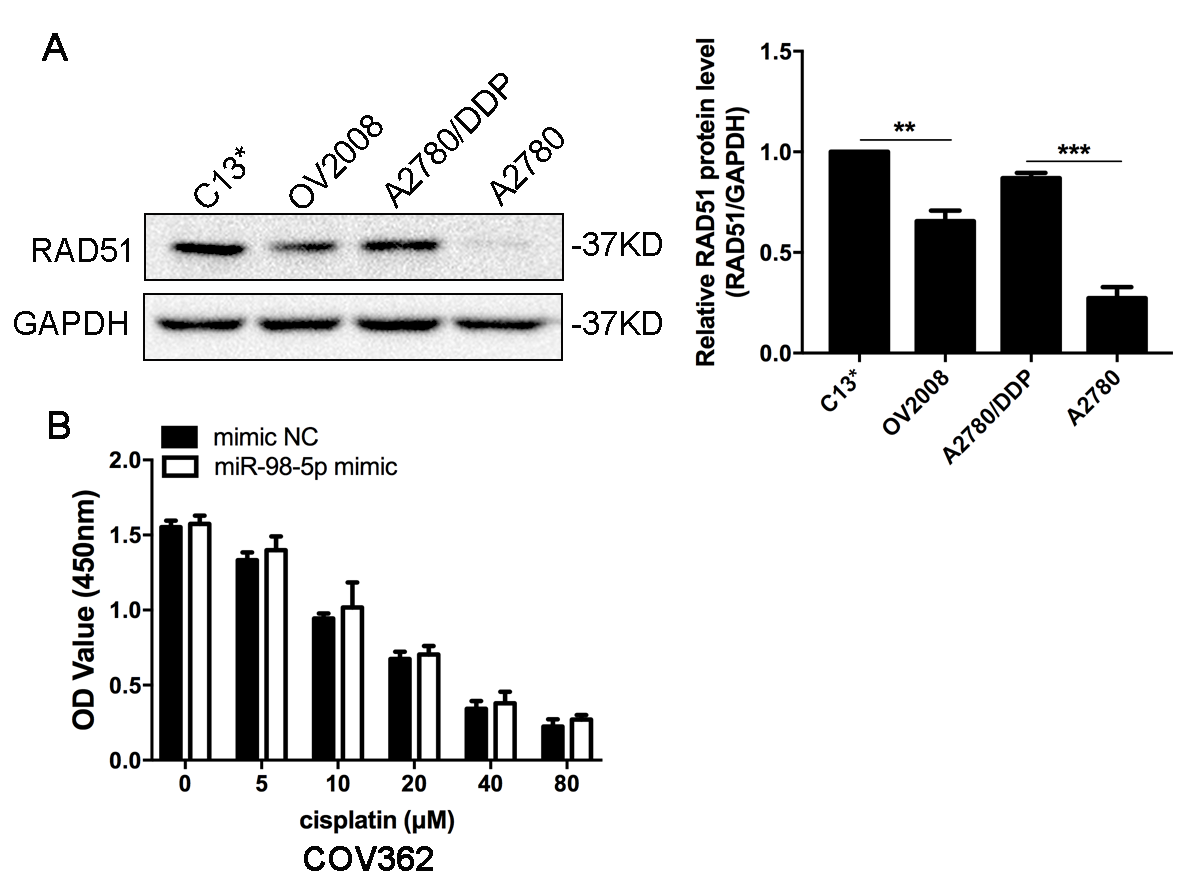


**Supplementary Figure 5**. (**A**) RAD51 protein levels in indicated cells were determined by western blot. (**B**) COV362 cells were transfected with mimic NC or miR-98-5p mimic for 24 h, and then cell viability was assayed after treatment with increasing concentrations of cisplatin for 48 h by CCK-8 assay. **p < 0.01; ***p < 0.001.
